# The impact of pazopanib and extremity radiotherapy on transaminase elevations in soft tissue sarcoma

**DOI:** 10.2340/1651-226X.2026.46196

**Published:** 2026-07-28

**Authors:** Bauke H.G. van Riet, Shermarke Hassan, Thomas R. de Wijkerslooth, Tom T.P. Seijkens, Alwin D.R. Huitema, Neeltje Steeghs, Rick L. Haas

**Affiliations:** a Department of Radiotherapy, the Netherlands Cancer Institute, Amsterdam, the Netherlands; b Department of Biometrics, the Netherlands Cancer Institute, Amsterdam, the Netherlands; c Department of Gastrointestinal Oncology, the Netherlands Cancer Institute, Amsterdam, the Netherlands; d Department of Medical Oncology, the Netherlands Cancer Institute, Amsterdam, the Netherlands; e Department of Pharmacy and Pharmacology, the Netherlands Cancer Institute, Amsterdam, the Netherlands; f Princess Máxima Center for Pediatric Oncology, Utrecht, the Netherlands; g Department of Clinical Pharmacy, University Medical Center Utrecht, Utrecht University, Utrecht, the Netherlands; h Department of Medical Oncology, University Medical Center Utrecht, Utrecht University, Utrecht, the Netherlands; i Department of Radiotherapy, Leiden University Medical Center, Leiden, the Netherlands

**Keywords:** Radiotherapy, pazopanib, transaminase elevations, soft-tissue sarcoma, hepatotoxicity

## Abstract

**Background and purpose:**

Unexpected high rates of grade ≥ 3 transaminase elevations were observed in patients receiving pazopanib and extremity radiotherapy. Although alanine transaminase (ALT) and aspartate transaminase (AST) primarily originate from the liver, both are also present in muscle tissue. In extremity soft tissue sarcomas (STS), where large muscle volumes are within the radiation field, radiation-induced muscle damage may contribute to these elevations. This study evaluated the impact of extremity radiotherapy, pazopanib, and their combination on the incidence and degree of ALT/AST elevations in STS patients.

**Patients and methods:**

A prospective cohort treated with extremity radiotherapy alone was combined with a retrospective cohort treated with pazopanib alone or in combination. Patient characteristics, treatment details, and laboratory values (liver enzymes and muscle injury biomarkers) were collected. Descriptive analyses and logistic regression were conducted to evaluate the association with grade ≥ 3 ALT/AST elevations.

**Results:**

Among 154 patients (10 radiotherapy, 108 pazopanib, 36 radiotherapy plus pazopanib), none of the radiotherapy-only patients experienced grade ≥ 3 elevations. Post-irradiation ALT/AST, creatine kinase and myoglobin elevations were minimal. Grade ≥ 3 elevations occurred in 6% of patients receiving pazopanib and 36% of those receiving both radiotherapy and pazopanib. All elevations typically resolved after pazopanib discontinuation. Grade ≥ 3 elevations were significantly associated with pazopanib, and modestly associated with pazopanib trough levels (odds ratio [OR]: 1.43 per 10 mg/L, 95% confidence interval [CI]: 1.12–1.90), but not with radiotherapy.

**Interpretation:**

Adding pazopanib to radiotherapy in localised extremity sarcoma significantly increases the rate of AST/ALT elevations. Extremity radiotherapy alone does not induce clinically relevant transaminase elevations. Grade ≥ 3 elevations were associated with pazopanib treatment. The mechanism underlying the substantially higher incidence of transaminase elevations with combined pazopanib and radiotherapy, compared with pazopanib alone, remains unknown.

## Graphical abstract

**Figure:**
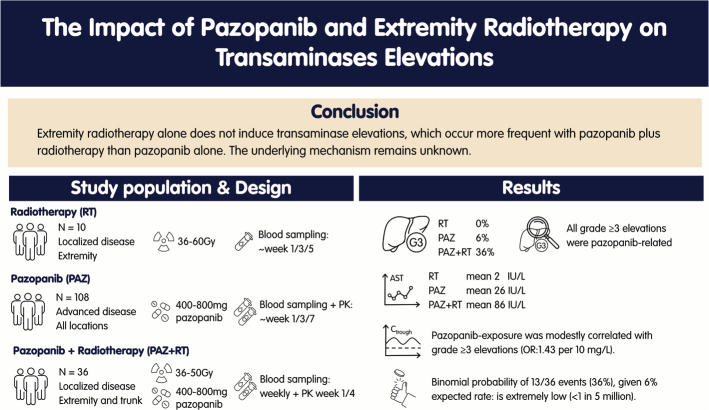

**HIGHLIGHTS**

- Extremity radiotherapy alone does not induce meaningful AST/ALT or muscle biomarker elevations.
- Grade ≥ 3 elevations occurred more frequently with pazopanib plus radiotherapy than with pazopanib alone.
- Severe AST/ALT elevations typically resolved after pazopanib discontinuation.
- Higher pazopanib C_trough_ modestly increased the risk of elevations, with no other factors identified.
- The reason why transaminase elevations occurred six times more often with pazopanib plus radiotherapy than with pazopanib alone remains not fully understood.

## Introduction

Soft tissue sarcomas (STS) are a heterogeneous group of rare cancers, accounting for approximately 1% of all newly diagnosed cancers [1]. Surgery is the cornerstone of treatment for localised STS and is often combined with preoperative radiotherapy to improve local control rates in deep-seated intermediate- and high-grade sarcomas. Despite these interventions, approximately 15% of patients develop local recurrences and 30% develop distant metastases [2]. As a result, various efforts have been made to improve treatment outcomes.

Two such efforts were the phase one PASART-1 trial (NCT01985295) [3] and phase two PASART-2 trial (NCT02575066) [4], which evaluated the angiogenesis inhibitor pazopanib combined with preoperative radiotherapy. While the combination was generally well tolerated, one-third of patients developed transient, isolated CTCAE grade ≥ 3 alanine transaminase (ALT) and aspartate transaminase (AST) elevations that required premature pazopanib discontinuation [5]. Although pazopanib is known to cause transaminase elevations, the reported incidence is substantially lower, suggesting additional contributing factors [6–10]. Unexpectedly, neither trial was able to identify a reasonable explanation for the high incidence of transaminase elevations.

Despite extensive clinical experience, the mechanism underlying pazopanib-associated transaminase elevations remains unclear. Clinically, elevations typically occur within 3 to 12 weeks and display a mixed pattern without immunoallergic or autoimmune features [11, 12]. Higher pazopanib exposure has been associated with increased AST and ALT levels, suggesting a partial exposure-dependent effect [13–16]. Although the liver is the primary source of these enzymes, ALT and AST are also present in muscle, kidney, and brain tissue [17–19]. In extremity STS, where tumours often involve large volumes of adjacent muscle, radiation-induced muscle damage could potentially contribute to these elevations.

Given these uncertainties, this study aimed to assess the impact of extremity radiotherapy, pazopanib treatment and exposure, and their combination on the incidence and degree of ALT and AST elevations in STS patients.

## Methods

### Study design

This study combined data from the PASART [3–4] trials with a prospective observational clinical study and a retrospective record review, conducted at the Netherlands Cancer Institute. Three comparative cohorts were included: (1) a prospective hospital-based radiotherapy cohort (RT); (2) a retrospective hospital-based pazopanib cohort (PAZ); and (3) patients enrolled in the PASART trials receiving concurrent pazopanib and radiotherapy (PAZ+RT).

The prospective study was approved by the local medical ethics committee (NedMec-O:23-007) and the retrospective record review by the institutional review board (IRBd25-019). All PASART patients provided consent for the use of their data.

### Patient population

Eligible patients were aged ≥18 years with histologically confirmed STS. Exclusion criteria across cohorts included Ewing sarcoma or other PNET family tumours, rhabdomyosarcomas, impaired organ function, liver disease, or WHO performance status (WHO PS) ≥ 2, to yield a comparable patient population to the original PASART participants.

The two hospital-based cohorts included: (1) a prospective RT cohort of patients treated in 2023–2024 with localised, non-metastatic extremity STS receiving neo-adjuvant or adjuvant radiotherapy, and (2) a retrospective PAZ cohort of patients treated with pazopanib monotherapy between 2011 and 2025. Cohort-specific exclusion criteria included prior or concurrent systemic therapy for RT, and concurrent systemic therapy or radiotherapy, missing laboratory values, lack of consent for use of data, or pazopanib discontinuation within 4 weeks for reasons unrelated to toxicity for PAZ.

Patients in the PAZ+RT cohort received neo-adjuvant radiotherapy with concurrent once-daily pazopanib for localised non-metastatic STS of the extremities and trunk between 2011 and 2018 as part of the PASART trials. Pazopanib was initiated 1 week prior to radiotherapy and continued until completion of radiotherapy. PASART-1 enrolled 11 patients across three dose levels: 400 mg (*n* = 3), 600 mg (*n* = 4), and 800 mg (*n* = 4), all receiving radiotherapy with 50 Gy in 25 fractions. PASART-2 included 25 patients receiving 800 mg pazopanib with either 50 Gy in 25 fractions (*n* = 21) or 36 Gy in 18 fractions (*n* = 4).

### Data collection

RT cohort patients were identified following multidisciplinary team meetings, while PAZ cohort patients were identified through the hospital pharmacy prescription information system. Relevant clinical and treatment data were extracted from electronic healthcare records for both cohorts. PAZ+RT cohort data were obtained from the original PASART electronic case report forms and supplemented with healthcare record data.

Patient characteristics, pazopanib dosing and trough levels, radiotherapy details, and laboratory values were collected for all patients. Baseline patient characteristics included age, gender, tumour size, tumour grade, histological subtype, location, history of liver disease, and WHO PS. Radiotherapy details included treatment schedule, delivered dose, and treated volumes. Laboratory values included AST and ALT, and additional biomarkers to differentiate between liver and muscle damage: creatine kinase, myoglobin, serum albumin, gamma-glutamyl transpeptidase (GGT), alkaline phosphatase (ALP), total bilirubin, lactate dehydrogenase (LDH), and blood coagulation time.

**Figure 1:**
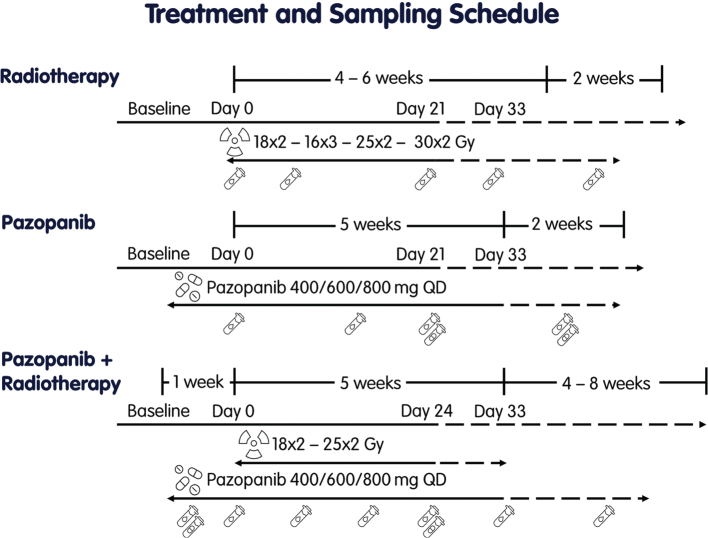
Treatment and sampling schedule cohorts, sampling collection times are indicated by collection vials, showing both laboratory and pharmacokinetic sampling points.

Blood sampling schedules differed per cohort: RT patients were sampled at baseline, end of week 1 (day 5), end of week 3 (day 19), and the last day of radiation (day 18, 25, or 33, depending on treatment schedule). On days 5 and 19, samples were collected both immediately prior and 2 h after radiation. For the PAZ and PAZ+RT cohorts, laboratory values were collected from treatment initiation through 8 weeks, with weekly sampling in PAZ+RT according to the PASART protocol and variable sampling in PAZ reflecting routine clinical care. An overview of treatment schedules and sampling times for all cohorts is shown in Figure 1.

### Transaminase elevations

Transaminase elevations were assessed during the first 8 weeks following treatment initiation to minimise confounding and align with PASART trial timelines. Elevations were graded according to the CTCAE version 5.0, and were categorised as either grade < 3 or ≥ 3. Elevations were further classified as muscle- or liver-related based on laboratory biomarkers, the AST/ALT ratio, with a predominance of AST relative to ALT considered supportive of a muscle-derived origin, and evaluated according to Hy’s law [20]. In the RT cohort, CK and myoglobin were used to support identification of a muscle-derived origin. In the PAZ and PAZ+RT cohorts, classification relied primarily on the treating physician’s assessment, supplemented by GGT, ALP, bilirubin, LDH, and CK when available. Among patients with grade ≥ 3 liver-related elevations, the R ratio and the treating physician’s assessment were used to determine the pattern of injury as hepatocellular (*R* > 5), mixed (R 2–5), or cholestatic (*R* < 2), and likelihood of pazopanib-induced liver injury [18].

### Pharmacokinetics

Pazopanib plasma trough levels were measured during standard clinical care (PAZ cohort) or within PASART-2 (PAZ+RT cohort). Trough levels were extrapolated using the formula by Wang et al. and were analysed using a validated liquid chromatography-tandem mass spectrometry (LC-MS/MS) assay [21, 22]. To ensure accurate estimation of trough levels, samples were required to be collected at least 3.5 h after dosing, and within 43 (twice daily) or 55 (once daily) h post-dose, as extrapolation beyond the dosing interval plus one half-life was considered unreliable.

In the PAZ cohort, trough samples were generally collected at weeks 4 and 8. Patient-level exposure was estimated by calculating the geometric mean of all available samples. In PAZ+RT, trough samples were collected on day 1 and day 22, with exposure based on day 22 given its steady-state condition. Cohort-level exposure was defined as the median patient-level exposure within each cohort.

### Statistical analysis

Summary tables for continuous variables include median and interquartile range. For categorical variables, counts and proportions are reported. Between-cohort differences were assessed using Fisher’s exact test, unpaired t-tests, or one-way analysis of variance (ANOVA), as appropriate. Associations in plasma AST and ALT were visualised per cohort using generalised additive models with cubic regression splines.

The RT cohort was analysed first to assess the association between radiotherapy and transaminase elevations in the absence of pazopanib. Logistic regression analysis was performed to evaluate associations between cohorts, patient and treatment characteristics, and grade ≥ 3 transaminase elevations. No correction for multiple comparisons was applied.

Sensitivity analyses were conducted using a lower clinically relevant grade ≥ 2 threshold, aligning with the liver toxicity warning threshold of the U.S. Food and Drug Administration (FDA) and European Medicines Agency (EMA) [10, 23]. To assess whether the observed event rate in PASART trials exceeded the expected rate, a one-sided, one-sample binomial test was performed using reference event rates from the Summary of Product Characteristics (SmPC) and literature as reference ranges (2% to 11%) [6–10]. No imputation or analysis of missing data was performed. Analyses were performed in R version 4.4.2 [24]. *P*-values of < 0.05 were considered significant.

## Sample size radiotherapy cohort

Transaminase levels are generally stable, making significant elevations unlikely without intervention [25, 26]. Based on prior literature and the pazopanib SmPC, grade ≥ 3 elevations occur in 7% of patients, whereas 36% of PASART patients experienced grade ≥ 3 elevations [6–10]. Assuming an expected incidence of 15% (between 7% and 36%), a sample size of 10 patients provided 80% power at 5% significance to reject the null hypothesis of 0.5% (conservative estimate) if at least one patient developed grade ≥ 3 elevations.

## Results

Baseline patient and tumour characteristics of the three cohorts are summarised in Table 1. RT included 10 patients, PAZ 108, and PAZ+RT 36. The selection and identification of PAZ patients is illustrated in Supplementary Figure 1. Briefly, age and WHO PS were comparable across cohorts. Sarcoma locations and subtypes differed. PAZ+RT patients had larger sarcomas, received higher radiation doses, and had higher median pazopanib C_trough_.

**Table 1:** Patient and treatment characteristics.

	Radiotherapy (RT)	Pazopanib (PAZ)	Pazopanib + Radiotherapy (PAZ+RT)
	*N* = 10	*N* = 108	*N* = 36
Sex, *N* (%)			
Male	6 (60)	36 (33)	23 (64)
Female	4 (40)	72 (67)	13 (36)
Age at diagnosis (years), median (IQR)	60 (5)	63 (19)	58 (19)
WHO PS			
0–1	10 (100)	108 (100)	36 (100)
Tumour size median (range)^a^	6.0 (2.4–12.8)	NA	9.0 (2.8–21.1)
FNCLCC grade, *N* (%)^a^			
Grade I/II	7 (70)	NA	20 (56)
Grade III	3 (30)	NA	16 (44)
Histological subtype, *N* (%)			
USTS	5 (50)	25 (23)	19 (53)
Synovial sarcoma	0 (0)	12 (11)	2 (6)
Myxofibrosarcoma	0 (0)	4 (4)	9 (25)
MLS	3 (30)	1 (1)	1 (3)
LMS	1 (10)	43 (40)	0 (0)
Other	1 (10)	23 (21)	5 (14)
Tumour site, *N* (%)			
Lower extremity	10 (100)	20 (19)	24 (67)
Upper extremity	0 (0)	2 (2)	4 (11)
Thorax	0 (0)	14 (13)	8 (22)
Abdomen	0 (0)	68 (62)	0 (0)
Other	0 (0)	4 (4)	0 (0)
Radiation schedule, *N* (%)			
25 × 2 Gy neo-adjuvant	3 (30)	NA	32 (89)
18 × 2 Gy neo-adjuvant	3 (30)	NA	4 (11)
14 × 3 Gy neo-adjuvant	2 (20)	NA	0 (0)
30 × 2 Gy adjuvant	1 (10)	NA	0 (0)
16 × 3 Gy adjuvant	1 (10)	NA	0 (0)
GTV, cc median (range)^b^	51 (25–478)	NA	189 (17–746)
CTV, cc median (range)^b^	292 (92–2,025)	NA	696 (91–1,554)
PTV, cc median (range)^b^	632 (260–3,241)	NA	850 (286–3,364)
Pazopanib starting dose, *N* (%)			
800 mg	NA	100 (92)	29 (81)
600 mg	NA	2 (2)	4 (11)
400 mg	NA	4 (4)	3 (8)
200 mg	NA	2 (2)	0 (0)
Pazopanib prescription			
Discontinuation	NA	8 (7)	12 (33)
Temporary interruption (≥ 10 days)	NA	5 (5)	1 (3)
Dose decrease	NA	8 (7)	0 (0)
Dose increase	NA	4 (4)	0 (0)
Pazopanib geometric mean c_trough_ (mg/L) per patient, median (IQR); mean^c^	NA	32.9 (24.8); 33.2	35.8 (26.0); 43.8
Grade ≥ 3 transaminase elevations			
AST and/or ALT	0 (0)	7 (6)	13 (36)
AST	0 (0)	5 (5)	11 (31)
ALT	0 (0)	7 (6)	13 (36)
Baseline AST (IU/L) mean, (median, IQR)	31 (27, 4)	27 (25, 11)	22 (22, 7)
Baseline ALT (IU/L) mean, (median, IQR)	31 (26, 4)	24 (20, 15)	24 (20, 10)

AST: aspartate aminotransferase; ALT: alanine aminotransferase; CTV: clinical target volume; GTV: gross tumour volume; PTV: planning target volume; FNCLCC: Fédération Nationale des Centres de Lutte Contre le Cancer; IQR: interquartile range; WHO PS: World Health Organization performance status; NA: not applicable; MLS: myxoid liposarcoma; USTS: undifferentiated soft tissue sarcoma; LMS: leiomyosarcoma (excluding skin).

a Sarcoma size and grade were not collected for the pazopanib cohort due to the metastatic setting.

b Radiotherapy treatment details were available for 19/36 PASART patients.

c Trough levels were available for 87/108 pazopanib patients and 24/36 PASART patients.

Transaminase elevations were absent in the RT cohort. In the PAZ cohort, seven of 108 patients (6%) developed grade ≥ 3 elevations and 15 of 108 (14%) developed grade ≥ 2 elevations. In PAZ+RT, 13 of 36 patients (36%) developed grade ≥ 3 and 16 of 36 (44%) grade ≥ 2 elevations. All grade ≥ 2 cases (*n* = 31) were attributed to pazopanib by the treating physician; 23 were classified as hepatocellular and eight as mixed pattern. No clinically apparent differences in pazopanib exposure, or biochemical parameters were observed between the two groups. None met Hy’s law criteria. A liver biopsy was performed in only one out of 20 patients with grade ≥ 3 elevations and showed moderately active chronic hepatitis with cholangitis, cholestasis, and mild mononuclear infiltration.

### Proportion of patients with grade ≥ 3 transaminase elevations

Given the higher incidence observed in the PAZ+RT cohort (PASART trials), the probability of these events was assessed relative to the PAZ cohort and literature-reported rates of 2% to 11% [6–10]. Assuming a true event rate of 6.5% (7/108), the probability of observing ≥ 13 events (36%) in 36 patients was < 1 in 5 million. For comparison, if the true event rate was 2% or 11%, the corresponding probabilities would be < 1 in 8 trillion and < 1 in 14,670, respectively. The observed incidence far exceeds what would be expected by chance given an expected event rate of 2% – 11%.

### Patterns of transaminase elevations

**Figure 2:**
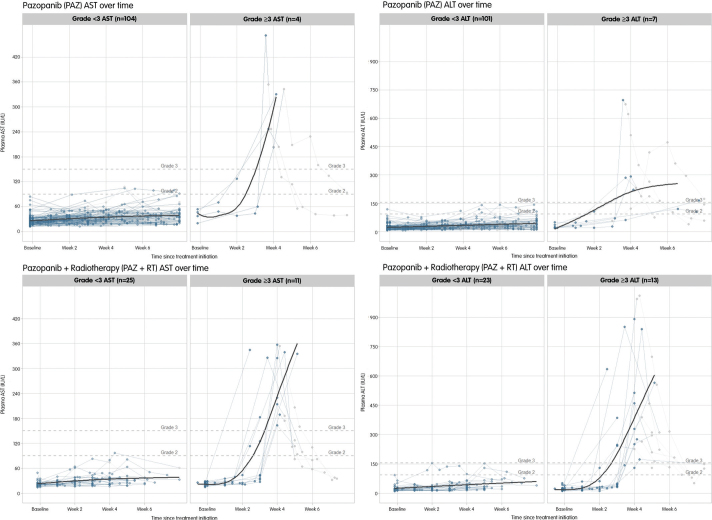
Plasma AST and ALT levels over time in patients receiving pazopanib, stratified by grade 3 elevations. Individual patient samples collected during pazopanib treatment are shown in blue; samples obtained after discontinuation are shown in grey. A trend line based on on-treatment samples is depicted in dark grey. Horizontal dashed lines indicate thresholds for CTCAE grade 2 and grade 3 toxicity.

The course of plasma AST and ALT levels is visualised in Figure 2 (PAZ and PAZ+RT) and Supplementary Figure 2 (RT). Transaminase elevations in the RT cohort were absent with mean AST and ALT increases of 2 IU/L (median: 3, range: 0–4) and 2 IU/L (median: 2, range: 0–7), respectively. Myoglobin and creatine kinase elevations were also negligible. No significant differences in laboratory biomarker levels were observed between pre- and post-radiation measurements.

In the PAZ and PAZ+RT cohorts, transaminase levels remained stable during the first 2 weeks but showed pronounced elevation in week 3. Levels typically normalised after pazopanib discontinuation. The patterns in PAZ+RT closely mirrored those observed in PAZ. Mean increases from baseline were 23 IU/L for AST (median: 11, interquartile range [IQR]: 20) and 89 IU/L for ALT (median: 19, IQR: 157) in PAZ and 33 IU/L for AST (median: 12, IQR: 22) and 190 IU/L for ALT (median: 44, IQR: 280) in PAZ+RT.

### Radiotherapy, pazopanib, and transaminase elevations

Firth’s penalised logistic regression was performed to assess the association between treatment and grade ≥ 3 transaminase elevations. Compared with PAZ+RT, both PAZ (odds ratio [OR]: 0.13, 95% confidence interval [CI]: 0.05–0.34) and RT (OR: 0.08, 95% CI: 0.01–0.74) were associated with significantly lower odds of grade ≥ 3 elevations (Table 2). These estimates should be interpreted with caution given the absence of grade ≥ 3 events in the RT cohort.

**Table 2:** Univariate logistic regression grade ≥ 2 and ≥ 3 transaminase elevations.

Covariate	Comparison	*N* (Grade ≥ 3 events)	Grade ≥ 3		Grade ≥ 2	
			OR (95% CI)	*p*	OR (95% CI)	*p*
Sex	Male vs. female	65 (8) vs. 89 (12)	0.90 (0.33–2.32)	0.830	1.16 (0.52–2.56)	0.710
Treatment^a^	PAZ vs. PAZ+RT	108 (7) vs. 36 (13)	0.13 (0.05–0.34)	< 0.001	0.21 (0.09–0.48)	< 0.001
	RT vs. PAZ+RT	10 (0) vs. 36 (13)	0.08 (< 0.01–0.74)	0.021	0.06 (< 0.01–0.52)	0.006
Age	per year	154 (20)	1.01 (0.98–1.06)	0.407	1.00 (0.97–1.03)	0.960
Pazopanib starting dose	< 800 mg vs. 800 mg	15 (3) vs. 129 (17)	0.60 (0.17–2.86)	0.473	0.73 (0.23–2.78)	0.610
Pazopanib geometric mean c_trough_	per 5 mg/L	111 (16)	1.20 (1.06–1.38)	0.007	1.14 (1.02–1.28)	0.023
	per 10 mg/L	111 (16)	1.43 (1.12–1.90)	0.007	1.30 (1.04–1.65)	0.023
Pazopanib geometric mean c_trough_	Q1 (< 20.6 mg/L) vs. Q4 (> 46.8 mg/L)	28 (1) vs. 28 (7)	10.80 (1.78–208.80)	0.031	3.95 (1.02–19.66)	0.061
Baseline AST	< 30 vs. ≥ 30	118 (15) vs. 36 (5)	0.91 (0.32–2.98)	0.870	0.69 (0.29–1.75)	0.420
Baseline ALT	< 31 vs. ≥ 31	121 (16) vs. 31 (4)	1.02 (0.34–3.77)	0.975	0.44 (0.18–1.09)	0.067

AST: aspartate aminotransferase; ALT: alanine aminotransferase; OR: odds ratio; CI: confidence interval.

a Odds ratios for treatment were estimated using Firth penalised logistic regression due to no events in the RT cohort.

**Figure 3:**
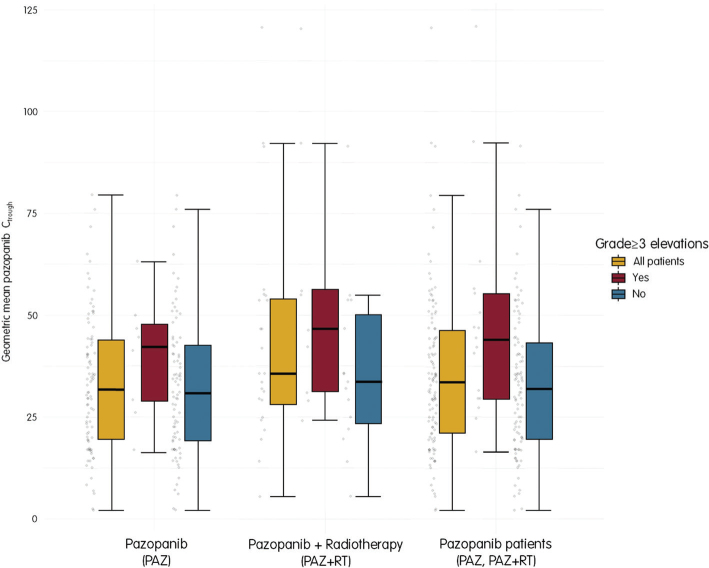
Geometric mean pazopanib trough concentrations per cohort, stratified by grade 3 elevations. Individual patients are shown in grey.

Among pazopanib-treated patients, median C_trough_ was slightly higher in the PAZ+RT cohort than in the PAZ cohort (35.8 vs. 32.9 mg/L) (Figure 3). Within each cohort, patients with grade ≥ 3 elevations had higher median C_trough_ than those without (PAZ+RT, 47 vs. 34 mg/L; PAZ, 44 vs. 31 mg/L). Higher C_trough_ was associated with an increased risk of grade ≥ 3 elevations (OR: 1.43 per 10 mg/L; 95% CI: 1.12–1.90), consistent with a modest exposure-toxicity relationship (Table 2). In multivariate analysis, the association between PAZ+RT and grade ≥ 3 elevations was partially attenuated but remained significant when adjusted for C_trough_ (OR: 5.44, 95% CI: 1.66–18.24) (Supplementary Table 1).

Descriptive analyses of pooled pazopanib-treated cohorts stratified by grade ≥ 3 transaminase elevations were performed to assess the associations with pazopanib exposure and patient characteristics, as all elevations were attributed to pazopanib and none occurred in the RT cohort. Patients were generally comparable between groups, except for median C_trough_, which was significantly higher in patients with grade ≥ 3 elevations (46 vs. 32 mg/L, *p* = 0.01) and grade ≥ 2 elevations (42 vs. 32 mg/L, *p* = 0.02) (Table 3). Other laboratory variables did not differ between groups (data not shown). Evaluation of patients with grade ≥ 3 elevations did not reveal any apparent patterns, risk factors, or probable causes across clinical characteristics, biochemical parameters, or inflammatory parameters (Supplementary Table 2).

**Table 3:** Patient and treatment characteristics of pazopanib patients.

	Total	Grade < 3 elevations	grade ≥ 3 elevations
	*N* = 144	*N* = 124	*N* = 20
Cohort, *N* (%)			
Pazopanib (PAZ)	108 (75)	101 (81)	7 (35)
Pazopanib + Radiotherapy (PAZ+RT)	36 (25)	23 (19)	13 (65)
Sex, *N* (%)			
Male	59 (41)	51 (41)	8 (40)
Female	85 (59)	73 (59)	12 (60)
Age at diagnosis (years), median (IQR)	61 (19)	60 (18)	63 (18)
WHO PS			
0–1	144 (100)	124 (100)	20 (100)
Tumour size PASART median (range)^a^	9.0 (2.8–21.1)	9.9 (4.5–20.8)	8.25 (2.8–21.1)
FNCLCC grade PASART, *N* (%)^a^			
Grade I/II	20 (56)	13 (57)	7 (54)
Grade III	16 (44)	10 (43)	6 (46)
Histological subtype, *N* (%)			
USTS	44 (31)	38 (31)	6 (30)
Synovial sarcoma	14 (10)	13 (10)	1 (5)
Myxofibrosarcoma	13 (9)	9 (7)	4 (20)
MLS	2 (1)	1 (1)	1 (5)
LMS	43 (29)	37 (30)	6 (30)
Other	28 (19)	26 (21)	2 (10)
Tumour site, *N* (%)			
Extremity	50 (35)	39 (32)	11 (55)
Thorax	22 (15)	19 (15)	3 (15)
Abdomen	68 (47)	62 (50)	6 (30)
Other	4 (3)	4 (3)	0 (0)
Pazopanib geometric mean c_trough_ (mg/L) per patient, median (IQR); mean^b^	33.8 (26.2); 35.6	32.0 (24.2); 33.2	45.5 (24.7); 49.3
Baseline AST IU/L mean, (median, IQR)	26 (24, 10)	26 (23, 10)	26 (25, 9)
Baseline ALT IU/L mean, (median, IQR)	24 (20, 13)	24 (20, 14)	25 (22, 8)

AST: aspartate aminotransferase; ALT: alanine aminotransferase; FNCLCC: Fédération Nationale des Centres de Lutte Contre le Cancer; IQR: interquartile range; WHO PS: World Health Organization performance status; MLS: myxoid liposarcoma; USTS: undifferentiated soft tissue sarcoma; LMS: leiomyosarcoma (excluding skin).

a Sarcoma size and grade were not collected for the pazopanib cohort due to the metastatic setting.

b Trough levels were available for 95/124 grade ≤ 2 patients and 16/20 grade ≥ 3 patients.

## Discussion

In this multi-cohort study, we evaluated the impact of extremity radiotherapy, pazopanib, and their combination on the incidence and degree of ALT or AST elevations in STS patients. This analysis was initiated following the unexpectedly high incidence of ALT or AST elevations observed in the PASART trials, in which patients with extremity STS received pazopanib concurrently with radiotherapy [3, 4]. Surprisingly, transaminase elevations occurred six times more often in patients treated with the combination of pazopanib and radiotherapy (36%) than pazopanib alone (2% – 11%). For this reason, we studied whether this difference could be explained by chance; however, that was proven very unlikely, with an estimated probability of < 1 in 14,670 or lower.

Given that the main difference between PAZ and PAZ+RT cohorts was the addition of extremity radiotherapy, we assessed the effect of extremity radiotherapy alone. Radiotherapy alone induced negligible changes in AST, ALT, and muscle-derived enzymes, indicating that radiation-induced muscle damage does not contribute meaningfully to transaminase elevations. These findings suggest that the observed elevations are most likely driven by pazopanib. However, these findings do not explain why transaminase elevations occurred six times more often in patients treated with the combination of pazopanib and radiotherapy, compared to pazopanib alone. Therefore, we investigated differences in pazopanib treatment schedule, clinical characteristics and patient-related factors, pazopanib pharmacokinetics and pharmacogenetics, and other potential factors.

Consistent with its known hepatotoxic potential, pazopanib treatment was associated with transaminase elevations. Affected patients typically exhibited stable transaminase levels during the first 2–3 weeks, followed by an abrupt increase that resolved upon pazopanib discontinuation, closely mirroring previously reported observations and supporting pazopanib as a probable cause [7]. Assessment using the Naranjo Adverse Drug Reaction Probability Scale similarly classifies pazopanib as a probable cause, supporting its role in these elevations [27].

To explore potential patient-related factors, we evaluated clinical characteristics and pazopanib exposure. Evaluation of patients with grade ≥ 3 elevations did not reveal any explanatory factors across clinical characteristics, biochemical parameters, or inflammatory parameters. Higher pazopanib C_trough_ values were associated with increased risk of grade ≥ 2 and grade ≥ 3 elevations. However, the exposure-toxicity relationship was modest, with relatively small median C_trough_ differences between cohorts. In multivariate logistic regression, the association between PAZ+RT and grade ≥ 3 elevations remained significant after adjusting for C_trough_, indicating that pazopanib exposure only partially explains the observed transaminase elevations.

Pazopanib-induced liver injury resembles an idiosyncratic drug-induced liver injury, likely arising from a complex interaction between its hepatic metabolism, formation of reactive intermediates, and downstream cellular stress responses [28, 29]. Pazopanib is predominantly metabolised by CYP3A4, with contributions from CYP1A2 and related enzymes, generating reactive metabolites that induce oxidative stress and mitochondrial dysfunction [29]. These metabolites have been associated with elevated transaminases and oxidative markers and may contribute to cellular dysfunction [28]. Endoplasmic reticulum stress signalling has also been identified as a contributing mechanism in pazopanib-induced hepatocyte injury [30]. Interpatient variability in drug metabolism may further influence the risk of hepatotoxicity [31]. While these mechanisms may explain how pazopanib induces transaminase elevations, they do not explain why the addition of radiotherapy would result in a higher frequency of transaminase elevations. Therefore, other mechanisms or additional patient-related factors may be involved.

Immune-mediated mechanisms may also play a role, as similar mechanisms have been described for several tyrosine kinase inhibitors like crizotinib, lapatinib, and imatinib [12]. If similar mechanisms apply to pazopanib, radiotherapy could theoretically lower the immunologic threshold through radiation-induced immune stimulation, thereby increasing the likelihood of autoantibody formation or cytotoxic immune responses against hepatocytes. Inflammatory infiltrates were observed in liver biopsies in case reports of severe pazopanib-induced hepato-toxicity, and corticosteroids have effectively been used to treat pazopanib-associated hepatotoxicity [32–34]. However, such inflammatory infiltrates may reflect a reactive response rather than true immune-mediated hepatotoxicity. Consistent with this, previous studies and clinical experience indicate that pazopanib-associated liver injury is generally self-limiting following discontinuation, even in the absence of corticosteroid therapy [32]. In our study, patients with grade ≥ 3 elevations lacked obvious haematological inflammatory markers, and the timing and pattern of these elevations were inconsistent with the typical onset time of immune-mediated hepatotoxicity, which generally occurs ≥ 6 weeks after treatment initiation [35, 36]. Collectively, this suggests that immune-mediated mechanisms are unlikely to explain the observed elevations.

Pharmacogenetic analyses suggest that certain genetic variants may contribute to the risk of pazopanib-induced hepatotoxicity. For example, HLA-B*57:01 carriers are at higher risk, as pazopanib interacts with the antigen-binding cleft of HLA-B*57:01, altering antigen presentation and triggering T-cell responses. This mechanism is best characterised for abacavir, which commonly induces hypersensitivity with rash, fever, and occasionally multi-organ toxicity within 3 weeks after treatment initiation in HLA-B*57:01 carriers [37]. However, HLA-B*57:01 occurs in approximately 5% of West Europeans, making it unlikely to explain the high incidence observed in PASART [38]. Variants in the haemochromatosis gene may also increase the risk of transaminase elevations by increasing hepatic iron deposition and associated oxidative stress, resulting in increased susceptibility to pazopanib-induced liver injury. While genetic factors may contribute in individual patients, they are unlikely to fully account for the observed differences [37, 39].

Taken together, despite considering multiple pharma-cological, clinical, and genetic factors, the cause of the high incidence of pazopanib-induced transaminase elevations in combination with radiotherapy remains unclear.

Although beyond the scope of this study, the observed increase in transaminase elevations with concurrent pazopanib and radiotherapy has practical clinical implications. Transaminase elevations were transient, and real-world data suggest patients can safely restart pazopanib at their original dose following temporary interruption, supporting close monitoring of transaminase levels as a sufficient and pragmatic approach [32]. From a radiotoxicity perspective, the recently published European Society for Medical Oncology (ESMO)–European Society for Radiotherapy and Oncology (ESTRO) consensus indicates that pre-emptive interruption is generally unnecessary for low-dose palliative skin and musculoskeletal radiotherapy, and advises against its combination with (ultra)central lung Stereotactic Body Radiation Therapy (SBRT)/ Stereotactic Ablative Radiotherapy (SABR) when large blood vessels, main bronchi, or the oesophagus are in the high-dose region, suggesting that interruption may be required depending on the irradiated site [40].

Despite the insights provided, several limitations of this study must be acknowledged. First, the design combining one retrospective and two prospective studies with different populations may have introduced heterogeneity in patient characteristics that may not fully be accounted for. Second, laboratory values were collected at different time points across cohorts, and not all biomarkers were available in each cohort. Third, patient selection bias cannot be fully excluded, as PAZ+RT patients were treated in clinical trials for curable disease, whereas PAZ patients were in an advanced disease setting. Fourth, although the RT cohort was powered to detect clinically meaningful transaminase elevations based on prior estimates, the relatively small sample size may have limited the detection of rare or more subtle effects and does not fully exclude a modest contribution of radiotherapy to the observed transaminase elevations. Fifth, as no correction for multiple comparisons was applied, logistic regression results should be interpreted with appropriate caution. Sixth, detailed dosimetry analysis of irradiated muscle tissue was not feasible due to the study design and incomplete imaging data in the PAZ+RT cohort, potentially limiting interpretation. Seventh, the classification of transaminase elevations relied on laboratory biomarkers and treating physician assessment rather than liver biopsy, limiting the ability to distinguish the precise source and mechanism of injury. Lastly, several potential confounders could not be formally evaluated, as they were inherent to the study design or restricted to a single cohort.

## Conclusion

Adding pazopanib to radiotherapy in localised extremity sarcoma increases the rate of transaminase elevations significantly. Radiation-induced muscle damage does not meaningfully contribute to transaminase elevations. Grade ≥ 3 elevations were associated with pazopanib. The mechanism underlying the substantially higher observed incidence of transaminase elevations with pazopanib in combination with radiotherapy, compared to pazopanib alone, remains unknown.

## Supplementary Material



## Data sharing

Data used to support the findings of this study are available from the corresponding author upon request.

## References

[1] SEER*Explorer. SEER incidence data soft tissue [Internet]. National Cancer Institute; 2025 [cited 2025 Dec 24]. Available from: https://seer.cancer.gov/statistics-network/explorer/

[2] Blay JY, Le Cesne A, Penel N, Bompas E, Chevreau C, Duffaud F, et al. 1397O – the nationwide cohort of 26,883 patients with sarcomas treated in NETSARC reference network between 2010 and 2015 in France: major impact of multidisciplinary board presentation prior to 1st treatment. Ann Oncol. 2016;27:vi483. 10.1093/annonc/mdw388.03

[3] Haas RLM, Gelderblom H, Sleijfer S, van Boven HH, Scholten A, Dewit L, et al. A phase I study on the combination of neoadjuvant radiotherapy plus pazopanib in patients with locally advanced soft tissue sarcoma of the extremities. Acta Oncol. 2015;54:1195–201. 10.3109/0284186X.2015.1037404 25920360

[4] van Meekeren M, Bovee JVMG, van Coevorden F, van Houdt W, Schrage Y, Koenen AM, et al. A phase II study on the neo-adjuvant combination of pazopanib and radiotherapy in patients with high-risk, localized soft tissue sarcoma. Acta Oncol. 2021;60:1557–64. 10.1080/0284186X.2021.1971294 34554030

[5] Van Riet BHG, Van Meekeren M, Fiocco M, Miah A, De Pree I, Wiltink LM, et al. Long-term survival of participants in the PASART-1 and PASART-2 trials of neo-adjuvant pazopanib and radiotherapy in soft tissue sarcoma. Acta Oncol. 2025;64:69-77. 10.2340/1651-226X.2025.42333 39813174 PMC11758146

[6] van der Graaf WTA, Blay J-Y, Chawla SP, Kim D-W, Bui-Nguyen B, Casali PG, et al. Pazopanib for metastatic soft-tissue sarcoma (PALETTE): a randomised, double-blind, placebo-controlled phase 3 trial. Lancet. 2012;379:1879–86. 10.1016/S0140-6736(12)60651-5 22595799

[7] Powles T, Bracarda S, Chen M, Norry E, Compton N, Heise M, et al. Characterisation of liver chemistry abnormalities associated with pazopanib monotherapy: a systematic review and meta-analysis of clinical trials in advanced cancer patients. Eur J Cancer. 2015;51:1293–302. 10.1016/j.ejca.2015.03.019 25899987 PMC7451810

[8] Kapadia S, Hapani S, Choueiri TK, Wu S. Risk of liver toxicity with the angiogenesis inhibitor pazopanib in cancer patients. Acta Oncol. 2013;52:1202–12. 10.3109/0284186X.2013.782103 23594201

[9] Nakamura T, Matsumine A, Kawai A, Araki N, Goto T, Yonemoto T, et al. The clinical outcome of pazopanib treatment in Japanese patients with relapsed soft tissue sarcoma: a Japanese Musculoskeletal Oncology Group (JMOG) study. Cancer. 2016;122:1408–16. 10.1002/cncr.29961 26970174 PMC5069581

[10] European Medicines Agency. VOTRIENT (pazopanib): summary of product characteristics [Internet]. 2021 [cited 2025 Dec 24]; 60 p. Available from: https://www.ema.europa.eu/en/documents/product-information/votrient-epar-product-information_en.pdf

[11] Wang X, Chen R, Liu J, Wang E, Luo H. Liver injury related to vascular endothelial growth factor tyrosine kinase inhibitors: a pharmacovigilance analysis of the USA FDA adverse event reporting system (FAERS) database. Expert Opin Drug Saf. 2026;25(7):1351-59. 10.1080/14740338.2025.2460449 39881499

[12] LiverTox: clinical and research information on drug-induced liver injury [Internet]. Bethesda, MD: National Institute of Diabetes and Digestive and Kidney Diseases; 2012 [cited 2025 Dec 24]. Consulted entries: pazopanib, imatinib, lapatinib, crizotinib. Available from: https://www.ncbi.nlm.nih.gov/books/NBK547852/31643176

[13] Tan Z, Völler S, Yin A, Rieborn A, Gelderblom H, van der Hulle T, et al. Model-informed dose optimization of pazopanib in real-world patients with cancer. Clin Pharmacokinet. 2025;64:715–28. 10.1007/s40262-025-01504-5 40263237 PMC12064635

[14] Noda S, Yoshida T, Hira D, Murai R, Tomita K, Tsuru T, et al. Exploratory investigation of target pazopanib concentration range for patients with renal cell carcinoma. Clin Genitourin Cancer. 2019;17:e306–13. 10.1016/j.clgc.2018.12.001 30598361

[15] Suttle AB, Ball HA, Molimard M, Hutson TE, Carpenter C, Rajagopalan D, et al. Relationships between pazopanib exposure and clinical safety and efficacy in patients with advanced renal cell carcinoma. Br J Cancer. 2014;111:1909–16. 10.1038/bjc.2014.503 25349968 PMC4229638

[16] Minot-This MS, Boudou-Rouquette P, Jouinot A, de Percin S, Balakirouchenane D, Khoudour N, et al. Relation between plasma trough concentration of pazopanib and progression-free survival in metastatic soft tissue sarcoma patients. Pharmaceutics. 2022;14:1234. 10.3390/pharmaceutics14061224 35745797 PMC9231369

[17] Moriles KE, Azer SA. Alanine amino transferase. Treasure Island, FL: StatPearls Publishing; 2020 [cited 2025 Dec 24]. Available from: https://www.ncbi.nlm.nih.gov/books/NBK559278/

[18] Kwo PY, Cohen SM, Lim JK. ACG clinical guideline: evaluation of abnormal liver chemistries. Am J Gastroenterol. 2017;112:18–35. 10.1038/ajg.2016.517 27995906

[19] Lecina M, Castellar C, Pradas F, López-Laval I. 768-km multi-stage ultra-trail case study-muscle damage, biochemical alterations and strength loss on lower limbs. Int J Environ Res Public Health. 2022;19:876. 10.3390/ijerph19020876 35055697 PMC8776162

[20] Temple R. Hy’s law: predicting serious hepatotoxicity. Pharmaco-epidemiol Drug Safety. 2006;15:241-43. 10.1002/pds.1211 16552790

[21] Wang Y, Chia YL, Nedelman J, Schran H, Mahon FX, Molimard M. A therapeutic drug monitoring algorithm for refining the imatinib trough level obtained at different sampling times. Ther Drug Monit. 2009;31:579–84. 10.1097/FTD.0b013e3181b2c8cf 19730279

[22] Herbrink M, de Vries N, Rosing H, Huitema AD, Nuijen B, Schellens JH, et al. Quantification of 11 therapeutic kinase inhibitors in human plasma for therapeutic drug monitoring using liquid chromatography coupled with tandem mass spectrometry. Ther Drug Monit. 2016;38:649–56. 10.1097/FTD.0000000000000349 27749781

[23] U.S. Food and Drug Administration. VOTRIENT (pazopanib) prescribing information [Internet]. 2024 [cited 2025 Dec 24]. Available from: https://www.accessdata.fda.gov/drugsatfda_docs/label/2024/022465s036lbl.pdf

[24] R Core Team. R: a language and environment for statistical computing. Vienna, Austria: R Foundation for Statistical Computing; 2021. Available from: https://www.R-project.org/

[25] Miyake K, Miyake N, Kondo S, Tabe Y, Ohsaka A, Miida T. Seasonal variation in liver function tests: a time-series analysis of outpatient data. Ann Clin Biochem. 2009;46:377–84. 10.1258/acb.2009.008203 19641005

[26] Carobene A, Røraas T, Sølvik U, Sylte MS, Sandberg S, Guerra E, et al. Biological variation estimates obtained from 91 healthy study participants for 9 enzymes in serum. Clin Chem. 2017;63:1141–50. 10.1373/clinchem.2016.269811 28428356

[27] Naranjo CA, Busto U, Sellers EM, Sandor P, Ruiz I, Roberts EA, et al. A method for estimating the probability of adverse drug reactions. Clin Pharmacol Ther. 1981;30:239–45. 10.1038/clpt.1981.154 7249508

[28] Maillard M, Arellano C, Vachoux C, Chevreau C, Cabaton NJ, Pont F, et al. Biological role of pazopanib and sunitinib aldehyde derivatives in drug-induced liver injury. Metabolites. 2022;12:852. 10.3390/metabo12090852 36144257 PMC9505977

[29] Wang YK, Yang XN, Liang WQ, Xiao Y, Zhao Q, Xiao XR, et al. A metabolomic perspective of pazopanib-induced acute hepatotoxicity in mice. Xenobiotica. 2019;49:655–70. 10.1080/00498254.2018.1489167 29897827 PMC6628935

[30] Chen J, Zhu T, Deng Y, Chen J, Jiang G, He Q. Activation of HSPA5 contributes to pazopanib-induced hepatotoxicity through l-ornithine metabolism pathway and endoplasmic reticulum stress. J Pharm Pharmacol. 2025;77:564–81. 10.1093/jpp/rgae130 39673386

[31] Verheijen RB, Beijnen JH, Schellens JHM, Huitema ADR, Steeghs N. Clinical pharmacokinetics and pharmacodynamics of pazopanib: towards optimized dosing. Clin Pharmacokinet. 2017;56:987–97. 10.1007/s40262-017-0510-z 28185218 PMC5563343

[32] Westerdijk K, Krens SD, Steeghs N, van der Graaf WTA, Tjwa E, Westdorp H, et al. Real-world data on the management of pazopanib-induced liver toxicity in routine care of renal cell cancer and soft tissue sarcoma patients. Cancer Chemother Pharmacol. 2024;93:353–64. 10.1007/s00280-023-04615-7 38104304 PMC10951019

[33] Klempner SJ, Choueiri TK, Yee E, Doyle LA, Schuppan D, Atkins MB. Severe pazopanib-induced hepatotoxicity: clinical and histologic course in two patients. J Clin Oncol. 2012;30:e264–8. 10.1200/JCO.2011.41.0332 22802316

[34] Choi JW, Yoo JJ, Kim SG, Kim YS, Chin S. Pazopanib-induced severe acute liver injury: a case report. Medicine. 2021;100:e27731. 10.1097/MD.0000000000027731 34797298 PMC8601284

[35] Zhu B-k, Chen S-Y, Li X, Huang S-Y, Luo Z-Y, Zhang W. Real-world pharmacovigilance study of drug-induced autoimmune hepatitis from the FAERS database. Sci Rep. 2025;15:4783. 10.1038/s41598-025-89272-x 39922875 PMC11807099

[36] Czaja AJ. Drug-induced autoimmune-like hepatitis. Dig Dis Sci. 2011;56:958–76. 10.1007/s10620-011-1611-4 21327704

[37] Xu CF, Johnson T, Wang X, Carpenter C, Graves AP, Warren L, et al. HLA-B*57:01 confers susceptibility to pazopanib-associated liver injury in patients with cancer. Clin Cancer Res. 2016;22:1371–7. 10.1158/1078-0432.CCR-15-2044 26546620 PMC7444994

[38] Orkin C, Wang J, Bergin C, Molina J-M, Lazzarin A, Cavassini M, et al. An epidemiologic study to determine the prevalence of the HLA-B*5701 allele among HIV-positive patients in Europe. Pharmacogenet Genomics. 2010;20:307–14. 10.1097/FPC.0b013e3283390666 20375757

[39] Xu CF, Reck BH, Goodman VL, Xue Z, Huang L, Barnes MR, et al. Association of the hemochromatosis gene with pazopanib-induced transaminase elevation in renal cell carcinoma. J Hepatol. 2011;54:1237–43. 10.1016/j.jhep.2010.09.028 21145803

[40] van Aken ESM, Devnani B, Prelaj A, Castelo-Branco L, Marijnen CAM, Martins-Branco D, et al. ESMO-ESTRO consensus statements on the safety of combining radiotherapy with immune checkpoint inhibitors, VEGF(R) inhibitors, or multitargeted tyrosine kinase inhibitors. Ann Oncol. 2026;37(1):17–32. 10.1016/j.annonc.2025.09.008 41016600

