# Supplementary material for: The impact of pazopanib and extremity radiotherapy on transaminase elevations in soft tissue sarcoma
Source: Acta Oncol. 2026 Jul 28;65:46196. doi: 10.2340/1651-226X.2026.46196 (PMC13421152; doi:10.2340/1651-226X.2026.46196)
Supplement: Supplementary file 1 [file AO-65-46196-s1.pdf]

Supplementary material has been published as submitted. It has not been copyedited, or typeset by Acta Oncologica

The Impact of Pazopanib and Extremity Radiotherapy on Transaminase Elevations

Supplementary tables and Figures

- Supplementary Figure 1. Flow diagram of patient selection pazopanib cohort……………………………………..pg 2
- Supplementary Figure 2. Plasma transaminases over time radiotherapy cohort……………………………………pg 3
- Supplementary Table 1. Multivariate logistic regression grade ≥3 transaminase elevations.……………………pg 4
- Supplementary Table 2. Clinical characteristics of patients with grade ≥3 transaminase elevations…………pg 5

Supplementary Figure 1. Flow diagram of patient selection pazopanib cohort

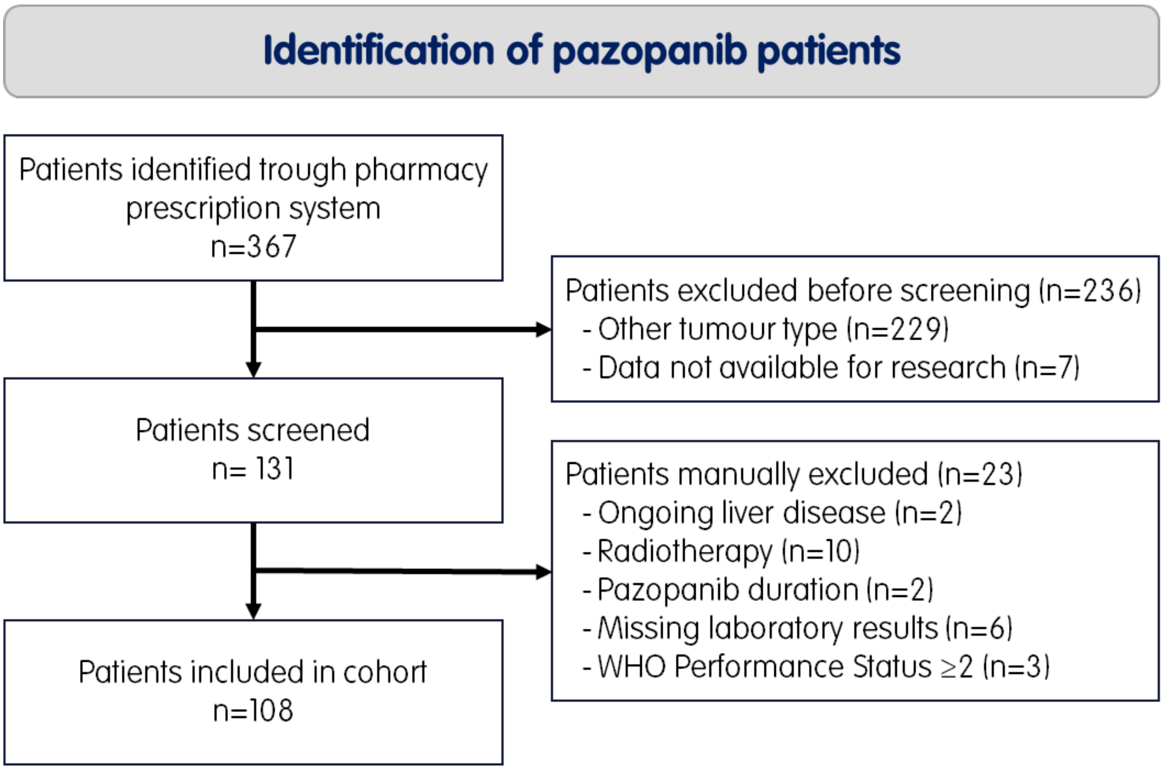

Supplementary Figure 1. Flow chart of patient identification and selection pazopanib monotherapy (PAZ) cohort.

Supplementary Figure 2. Plasma transaminases over time radiotherapy cohort

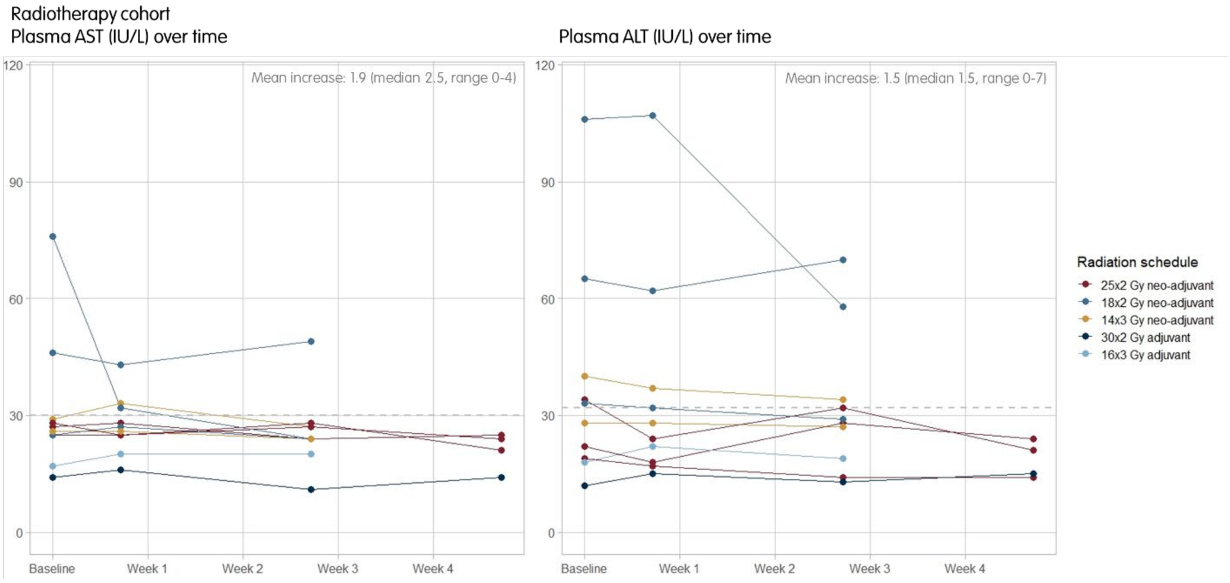

Supplementary Figure 2. Plasma AST and ALT levels over time in patients receiving radiotherapy. Horizontal dashed lines indicate thresholds for upper limit of normal.
AST, alanine aminotransferase; ALT, aspartate aminotransferase

Supplementary Table 1. Multivariate logistic regression grade ≥3 transaminase elevations

Univariate logistic regression

Covariate	Comparison	N (grade ≥3 events)	Grade ≥3 OR (95% CI)	*p-value*
Treatment	PAZ+RT vs PAZ	36 (13) vs 108 (7)	8.16 (3.01–23.92)	<0.001

Multivariate logistic regression

Covariate	Comparison	N (grade ≥3 events)	Grade ≥3 OR (95% CI)	*p-value*
Treatment	PAZ+RT vs PAZ	24 (9) vs 87 (7)	5.44 (1.66–18.24)	0.005
Pazopanib geometric mean c_{trough}	per 10 mg/L	111 (16)	1.34 (1.02–1.82)	0.045

Supplementary Table 2. Clinical characteristics of patients with grade ≥3 transaminase elevations

Cohort	Treatment & exposure			Toxicity Grade ≥3		Patient characteristics					Normal baseline laboratory liver parameters [a]				Co medication		Toxicity markers	
	Pazopanib dose (mg)	Radiotherapy schedule (fraction x Gy)	C_{trough} (mg/L)	AST	ALT	Age (years)	Sex	WHO PS	Tumor site	Liver metastases	AST (IU/L)	ALT (IU/L)	Bilirubin (µmol/L)	Albumin (g/L)	NSAID	Gastric suppressive agents	Notable inflammatory markers [b]	Hy's law
PAZ	800	NA	41	No	Yes	79	Female	0-1	Thorax	No	Yes	Yes	Yes	Yes	No	No	No	No
PAZ	400	NA	27	No	Yes	71	Female	0-1	Abdomen	Yes	Yes	Yes	Yes	Yes	No	No	No	No
PAZ	800	NA	63	No	Yes	64	Female	0-1	Abdomen	Yes	Yes	Yes	Yes	Yes	Yes	No	No	No
PAZ	800	NA	44	Yes	Yes	43	Male	0-1	Abdomen	Yes	No (47)	No (35)	Yes	Yes	No	Yes	No	No
PAZ	800	NA	51	Yes	Yes	61	Female	0-1	Abdomen	No	No (54)	No (50)	Yes	Yes	Yes	No	No	No
PAZ	800	NA	17	Yes	Yes	73	Female	0-1	Abdomen	No	No (37)	Yes	Yes	Yes	No	No	No	No
PAZ	800	NA	47	Yes	Yes	75	Male	0-1	Abdomen	Yes	Yes	Yes	Yes	Yes	No	No	No	No
PAZ+RT	800	25x2	121	No	Yes	39	Female	0-1	Extremity	No	Yes	Yes	Yes	Yes	No	No	No	No
PAZ+RT	800	25x2	24	No	Yes	44	Male	0-1	Extremity	No	Yes	Yes	Yes	Yes	No	No	No	No
PAZ+RT	800	25x2	47	Yes	Yes	72	Female	0-1	Extremity	No	Yes	Yes	Yes	Yes	No	No	No	No
PAZ+RT	800	25x2	31	Yes	Yes	61	Male	0-1	Extremity	No	Yes	Yes	Yes	Yes	No	No	No	No
PAZ+RT	800	25x2	92	Yes	Yes	50	Female	0-1	Extremity	No	Yes	Yes	Yes	Yes	Yes	No	No	No
PAZ+RT	800	25x2	NA	Yes	Yes	61	Male	0-1	Extremity	No	Yes	Yes	Yes	Yes	No	No	No	No
PAZ+RT	800	25x2	30	Yes	Yes	53	Female	0-1	Thorax	No	Yes	Yes	Yes	Yes	No	No	No	No
PAZ+RT	800	18x2	42	Yes	Yes	71	Female	0-1	Extremity	No	Yes	No (51)	Yes	Yes	No	No	No	No
PAZ+RT	600	25x2	NA	Yes	Yes	74	Male	0-1	Extremity	No	Yes	Yes	Yes	Yes	No	No	No	No
PAZ+RT	800	25x2	NA	Yes	Yes	78	Female	0-1	Extremity	No	Yes	Yes	Yes	Yes	No	No	No	No
PAZ+RT	800	25x2	56	Yes	Yes	71	Female	0-1	Extremity	No	Yes	Yes	Yes	Yes	Yes	No	No	No
PAZ+RT	600	25x2	NA	Yes	Yes	60	Male	0-1	Thorax	No	Yes	Yes	Yes	Yes	No	No	No	No
PAZ+RT	800	25x2	55	Yes	Yes	55	Male	0-1	Extremity	No	Yes	No (43)	Yes	Yes	No	No	No	No

AST, aspartate aminotransferase; ALT, alanine aminotransferase; C_{trough}, geometric mean pazopanib trough level; Gy, Gray; PAZ, pazopanib; PAZ+RT pazopanib + radiotherapy (PASART); WHO PS, World Health Organization performance status; NA, not applicable.

[a] Normal baseline laboratory parameters indicate laboratory values within the upper limit of normal at baseline, abnormal values are provide in parenthesis.

[b] Inflammatory markers include leukocytes and other notable inflammatory markers when available
